# Recent Advances in Nanopore Technology for Copper Detection and Their Potential Applications

**DOI:** 10.3390/nano13091573

**Published:** 2023-05-08

**Authors:** Alexander N. Vaneev, Roman V. Timoshenko, Petr V. Gorelkin, Natalia L. Klyachko, Alexander S. Erofeev

**Affiliations:** 1 Chemistry Department, Lomonosov Moscow State University, 119991 Moscow, Russia; 2 Research Laboratory of Biophysics, National University of Science and Technology “MISIS”, 119049 Moscow, Russia

**Keywords:** nanopore-based detection, copper ions, analytical chemistry, biological nanopores, solid-state nanopores, biological nanopores

## Abstract

Recently, nanopore technology has emerged as a promising technique for the rapid, sensitive, and selective detection of various analytes. In particular, the use of nanopores for the detection of copper ions has attracted considerable attention due to their high sensitivity and selectivity. This review discusses the principles of nanopore technology and its advantages over conventional techniques for copper detection. It covers the different types of nanopores used for copper detection, including biological and synthetic nanopores, and the various mechanisms used to detect copper ions. Furthermore, this review provides an overview of the recent advancements in nanopore technology for copper detection, including the development of new nanopore materials, improvements in signal amplification, and the integration of nanopore technology with other analytical methods for enhanced detection sensitivity and accuracy. Finally, we summarize the extensive applications, current challenges, and future perspectives of using nanopore technology for copper detection, highlighting the need for further research in the field to optimize the performance and applicability of the technique.

## 1. Introduction

Copper is a transition metal that plays an essential role in biological systems [1,2,3]. Copper is a cofactor for several enzymes involved in important physiological processes. Copper-based enzymes play important roles in various physiological processes, including pigmentation (tyrosinase), epigenetic modification (lysyl oxidase-like 2), respiration (cytochrome c oxidase), iron uptake (ceruloplasmin), antioxidant defense (Cu/Zn superoxide dismutase), neurotransmitter synthesis, and metabolism (dopamine β-hydroxylase) [4]. However, excessive copper intake or exposure can lead to toxicity and various health problems, including liver damage [5], neurodegenerative disorders [6,7,8], and Wilson’s disease [9]. The exposure of the human body to Cu^2+^ can occur through the consumption of contaminated water resulting from industrial or consumer waste. In view of the deleterious effects of such exposure, the Environmental Protection Agency acknowledges Cu^2+^ as a potential trace pollutant and stipulates a permissible limit of 20 μM of Cu^2+^ in drinking water [10].

Given the importance of copper in biological systems, there is a need for accurate and sensitive methods for the detection and monitoring of copper levels in water and biological samples, such as blood, urine, and cerebrospinal fluid [6,11]. Conventional methods for copper detection include colorimetric and fluorometric assays [12], electrochemical methods [13,14,15,16], and mass spectrometry [17]. Colorimetric and fluorescent sensors based on organic compounds have been intensively developed for the detection of Cu^2+^ ions [18]. However, these sensors typically suffer from low selectivity and poor water solubility. The main disadvantages are the inapplicability of colorimetric and fluorescent sensors to analyze levels of Cu^2+^ ions with dynamic changes in concentration and slow response times.

Conventional physical techniques for copper ion detection include atomic absorption or emission spectroscopy (AAS/AES) [19] and inductively coupled plasma mass spectrometry (ICP-MS) [20]. The techniques enable the sensitive and accurate detection of metal ions and are considered as standard methods. However, these methods often require complex sample preparation, expensive instrumentation, and skilled personnel. Moreover, these methods may not be suitable for in situ or real-time monitoring of copper in various environments [21]. Thus, an ideal sensor has the characteristics of easy development, fast detection, selectivity, good sensitivity, and reversibility. 

Nanopore technology is a rapidly developing area that has attracted much attention due to its potential applications in various fields, such as biosensing [22], genomics [23,24], and drug discovery [25]. This technology has many advantages, such as being label-free, ultrasensitive, possessing a high signal-to-noise ratio, having single-molecule resolution, and being able to detect and identify DNA [26], RNA [27], peptides [28], proteins [29], biomarkers, nanoparticles [30], and other small molecules [31]. Nanopore-based sensors have emerged as a promising alternative for the detection of copper ions [21] and other heavy metals [31]. Nanopores can be functionalized with specific molecules or materials that selectively interact with copper ions, enabling sensitive and label-free detection in real time. The most commonly used nanopore types for sensing applications are solid-state and biological nanopores [32]. Biological nanopores such as α-hemolysin have been used for the detection of copper ions [33,34]. Solid-state and hybrid nanopores have also been explored for copper detection in biological fluids with promising results [35]. In general, the development of sensitive and selective nanopore-based sensors for the detection of copper could have important implications for the diagnosis and monitoring of copper-related diseases, as well as for the understanding of copper metabolism and regulation in biological systems.

Biological nanopores are protein channels that are naturally found in biological membranes, such as the α-hemolysin pore [36]. Similar to solid-state nanopores, biological nanopores can also be modified or engineered to have specific properties for sensing applications. It should be noted that biological nanopores are among the most common nanopores for the detection of metal ions [37].

**Figure 1:**
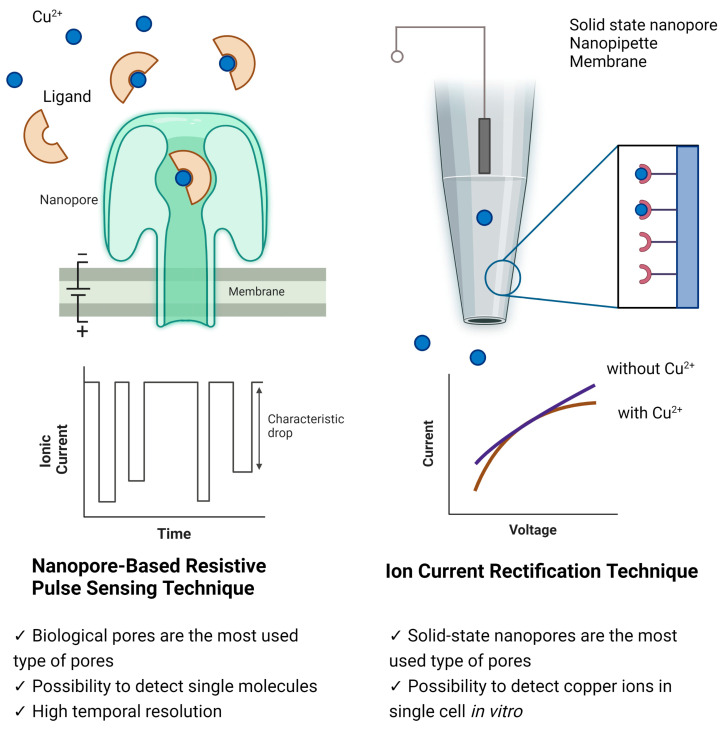
Summarized techniques and types of nanopores for copper detection.

Nanopore technology offers a promising platform for various sensing applications, including the detection of copper ions. To detect copper ions, there are two main techniques using nanopores: resistive pulse sensing and ion current rectification. The main principle of the first method is the use of molecules specific to copper ions, followed by the detection of complexes using a nanopore. The second principle is based on the modification of the nanopore channel by copper-binding ligands. After the binding of copper by ligands, the charge on the surface of the nanopore channel changes, and therefore the measured current–voltage characteristic changes (Figure 1). The ability to detect and monitor copper ions in real time using nanopore-based sensors could have significant implications in various fields, such as environmental monitoring, food safety, and medical diagnosis. Many good reviews have been written on various aspects of nanopore-based detection [21,31,32,38,39]. This review focused on recent advances in nanopore-based copper detection, highlighting the various types of nanopores and sensing strategies that have been developed. 

## 2. Techniques of Nanopore-Based Copper Detection

### 2.1. Nanopore-Based Resistive Pulse Sensing Technique

Nanopore-based sensors are a type of sensor that utilize nanoscale pores to detect and analyze different types of molecules [40,41,42,43]. Briefly, the fundamental principle of nanopore-based sensors is based on the analysis of the electrical current when molecules are passing through a nanopore. The basic setup of a nanopore-based sensor includes a nanoscale pore that should exactly correspond to the dynamic size of the target molecule/detection substance in the solution phase [44]. The nanoscale pore is positioned between two Ag/AgCl electrodes in electrolyte solution. With further application of electric potential to the described electrolytic cell, the ion current begins to flow through the nanopore. When a target molecule passes through the nanopore, it temporarily obstructs the flow of ions, causing a change in the measured current. The main analyzed current parameters of this process are the dwell time, amplitude of events, and event frequency. The significant part of the obtained data is unique for each molecule, which helps to determine the identity and properties of the analyte. The key features of nanopore-based sensors, highlighted in many studies/reviews, are real-time single molecule detection, potential for label-free detection, and a wide range of measurement conditions [31,45,46]. 

Despite their potential advantages, nanopore-based sensors also face challenges including the need to improve existing methods for the fabrication of biological and solid-state nanopores, signal-to-noise ratio optimization, and the development of suitable algorithms for data analysis [47,48,49]. Ongoing research aimed at solving the described problems and further improving the performance of nanopore-based sensors suggests that this scientific field is still promising for a wide range of practical applications [50].

### 2.2. Ionic Current Rectification Technique

Ionic current rectification (ICR) is a widely used technique for the detection of various types of analytes, including metals. Briefly, the main idea is to estimate the change in ion current as a result of the analyte molecular recognition event causing a conformational and/or surface charge variation inside the nanopore [51]. These described factors lead to a change in ion permeability or depletion of ions and hence to asymmetric current–voltage dependences [52,53]. As in resistive pulse nanopore-based sensing, the electrolytic cell consists of two electrodes located in the electrolyte on opposite sides of the nanopore. To achieve the maximum change in the surface charge of a nanopore, various modifiers are usually used to bind to the target analyte. Therefore, a lot of modern research is devoted to the search and use of new types of modifiers that strongly affect the sensitivity of this technique. A negative feature of the technique is the need to regenerate the sensor in a stronger complexing agent after a single measurement and at low scan rates, which imposes some restrictions on possible applications [54]. In addition, sensors using the ICR technique are predominantly suitable for measuring solutions.

However, despite these limitations, the ICR technique has proven to be a powerful tool for the detection of a wide range of analytes, including small molecules, proteins, and other biomolecules. The key feature of the ICR technique is the absence of the use ultra-small and low-conductivity nanopores, which results in a significant deterioration in the signal-to-noise ratio [43]. The technique is also attractive due to its simplicity and low cost compared to other analytical techniques. With ongoing advancements in nanopore fabrication, surface modification, and analytical methods, the ICR technique is expected to continue to gain popularity and be further developed for new and diverse applications in the future [39,55,56,57].

## 3. Applications of Nanopore-Based Sensors for Copper Detection

### 3.1. Nanopore-Based Resistive Pulse Sensing Technique

Recently, two types of nanopores have been used as nanopores: biological protein pores and synthetic nanopores. Protein nanopores are more reproducible and provide the highest resolution, while synthetic nanopores are more stable and robust [58]. 

Due to the small size of metal ions, it is impossible to detect them using unmodified nanopores or without additional sensitive probes. There are several strategies for detecting metal ions: (1) using biomolecules as carriers; (2) using a biological nanopore with a metal-sensitive site on the inner surface of the nanopore; and (3) using chemical reactions.

One of the first studies devoted to the detection of metals in solution was a work that used α-hemolysin mutant 4H containing histidine at specific positions. It was found that the signatures of events, such as the average blocking amplitude and/or translocation time of Zn^2+^, Co^2+^, and Cd^2+^ ions, were completely different, which made it possible to detect Zn^2+^ in the presence of other metal ion species and even achieved simultaneous detection of the three types of metal ions [59]. However, the use of this strategy has not yet received due development for the detection of copper ions.

The most-used strategy for the detection of copper ions using nanopores is the use of probes capable of chelating copper ions. Various molecules can be used as carriers, ranging from DNA molecules to polymers [31].

**Figure 2:**
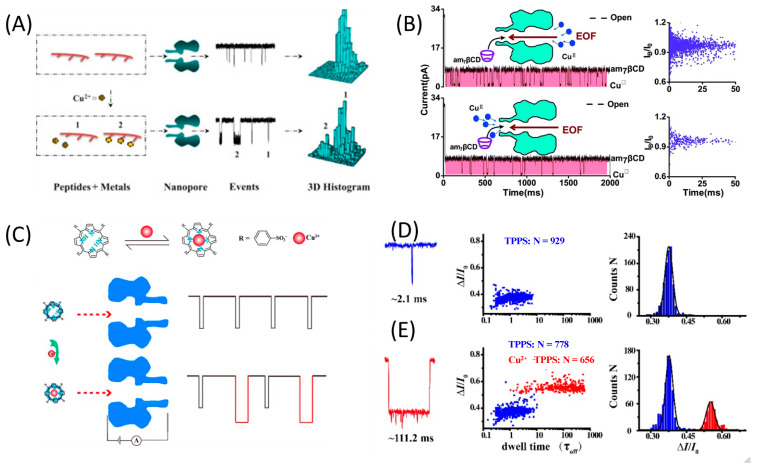
Nanopore pulse detection of Cu^2+^ ions using a chelating agent probe. (**A**) Scheme of detection of copper ions using polyhistidine probe. Reproduced from [33]. Copyright 2014 Elsevier (**B**) Detection of copper ions with βCD by α-hemolysin. Reproduced from [60]. Copyright 2017 Royal Society of Chemistry (**C**) Nanopore-based detection of Cu^2+^ using a TPPS as probe. The TPPS and Cu^2+^–TPPS complexes with the pore produced different signatures. (**D**) Expanded view of a typical event with TPPS, scatter plots of the events caused by TPPS, and histograms of normalized current blockade. (**E**) Expanded view of a typical event with TPPS + Cu^2+^, scatter plots of the events caused by TPPS (blue) and Cu^2+^–TPPS complexes (red), and histograms of normalized current blockage. Reproduced from [5].

In one study, it was demonstrated that the polyhistidine molecule can be used as a probe for the detection of Cu^2+^ ions based on a chelating reaction between them [33]. Without Cu^2+^ ions, interactions between copper chelating agents and protein pores lead to only one type of event (Figure 2A). After Cu^2+^ ions are added to the solution, they will interact with copper chelator molecules to form copper chelates, which will lead to events that have significantly different signs (e.g., residence time or blocking amplitudes) [3]. The peptides used included 3, 6, and 10 histidines. Despite the fact that the peptides possessed potential donor atoms, which, in turn, were effective chelators for various metal ions, the events corresponding to the Zn^2+^- and Cu^2+^-peptide complexes differed from each other in signal amplitude, which made it possible to distinguish them. The events of the Ni^2+^- and Co^2+^-peptide complexes had similar blocking amplitudes as that of the Cu^2+^-peptide chelate, although the residence times of their events were much shorter than that of the Cu^2+^-peptide complex. Thus, the authors managed to develop a specific method to distinguish copper ions from other transition metals with the LOD for Cu^2+^ of ~40 nM. 

Kang et al. used polyamine-decorated cyclodextrin as a recognition element for copper detection [60]. The strong binding affinity between Cu^2+^ and the amino groups of the cyclodextrin induced new current blockade events within the α-hemolysin pore. Three types of hemolysin protein nanopores, (WT)_7_, (M113R)_7_, and (M113F)_7_, were studied and significant differences in event characteristics were observed. In the case of (M113F)_7_, cyclodextrin almost permanently blocked the pore (77.2 ± 0.5%). It was noted that the (M113F)_7_ protein nanopore could provide increased resolution for recognition of Cu^2+^ ions compared to other types of hemolysin pores. The linear range for determining the Cu^2+^ concentration was 0.08–20 μM. The LOD was only 12 nM. The main advantage of this system was its high specificity for copper ions compared to other types of metal ions. The use of this sensor was also confirmed by the analysis of Cu^2+^ ions in running water (Figure 2B) [60].

Then, Kang et al. studied the interaction of CMβCD with Cu^2+^ in more detail [61]. It was noted that the Cu^2+^–CMβCD complex generated a distinctive signature that differed from that of the original βCD. It was shown that the chemical reaction between Cu^2+^ and CMβCD in the nanoreactor was completely different from that in the bulk solution. The formation constant increased in a nanopore by almost 15,000 times compared to in a bulk solution. The thermodynamic and kinetic constants of the Cu^2+^–CMβCD complex were affected by various experimental conditions, i.e., pH, transmembrane voltage, and temperature. It was also found that the binding of Cu^2+^ led to an increase in the channel current and not to blocking, as in the usual experiment with nanopores. It should be noted that in this work, the authors described in some detail the processes occurring in the hemolysin nanopore by studying the formation constant of Cu^2+^–CMβCD at various varied parameters. It was demonstrated for the first time that slightly acidic pH was favorable for the formation of a stable Cu^2+^–CMβCD complex. As the voltage between pores increased, the frequency of CMβCD binding to Cu^2+^ increased and the stability of the Cu^2+^–CMβCD complex decreased. It was shown that the process of complex formation was a spontaneous, endothermic process. This approach made it possible to evaluate the efficiency of metal ion chelation at the level of one molecule, which was a promising direction for the development of new drugs based on metal ion complexes.

In the following example, 5,10,15,20-tetrakis(4-sulfonatophenyl)-porphyrin (TPPS) was the ligand for copper ion chelation. Various chemosensors based on porphyrin were previously developed to detect heavy metal ions [62]. Kang et al. used a similar approach adopted in earlier fluorescent studies and nuclear medicine [5,63]. TPPS was used as the ligand for copper ion chelation. Nanopore-based detection of copper complexes was carried out using the α-hemolysin nanopore (Figure 2D). The signature of the Cu^2+^–TPPS complex was significantly different from that of free TPPS. The frequency of signature events showed a linear response for Cu^2+^ concentrations in the range of 0.03–1.0 µM (LOD 16 nM). The detection mechanism demonstrated excellent specificity towards Cu^2+^ ions and effectively distinguished them from other metal ions (Figure 2E). Moreover, the feasibility of implementing this approach in real scenarios was validated through the successful detection of Cu^2+^ ions in running water with recovery in the range of 95.0% to 101.7% [5].

One non-trivial approach for the determination of copper ions was taken by Mayne et al. [64]. Modified silicon dioxide nanoparticles by APTES were used to detect Cu^2+^ ions in solution. To determine the concentration of copper ions in the solution, the peak width was used to measure particle velocity, along with the magnitude and frequency of both resistive (Δi_r_) and conduction (Δi_c_) pulses, as illustrated in Figure 2D. The signal exhibited high specificity for Cu^2+^ in the presence of other metal ions, and its characteristics were modulated by adjusting the pH and ionic strength of the solution. This technique enabled the detection of Cu^2+^ ions at concentrations as low as 1 ppm with a brief 5 min incubation period and the capacity to accurately measure 10 ppm of Cu^2+^ in the presence of five other types of ions. Its potential application extends to the monitoring of heavy metals in both biological and environmental samples.

It is widely acknowledged that PrP is a protein that binds to copper, with the capability of absorbing numerous Cu^2+^ ions within its malleable N-terminal segment. In the next study, the prion peptide was immobilized through physical sorption onto the negatively charged walls of a nanopipette, and its functionalization was monitored in real time via chronoamperometry. The sensor utilized the unique properties of the PrP octarepit domain and could be regenerated multiple times by EDTA treatment without any significant loss in performance, demonstrating the stability of the interaction of the peptide with the nanopipette walls. However, the disadvantage of this method was its low sensitivity and selectivity [65].

For some chemical reactions, the presence of copper ions is crucial. For example, Cu^+^ catalyzes the 1,3-dipolar cycloaddition of azides to alkynes. This reaction belongs to the field of click chemistry and demonstrates the advantages of excellent ligation efficiency, high selectivity, and mild reaction conditions [66,67]. Therefore, this reaction underlies many methods of analysis, for example, electrochemical [68], colorimetric [69], and fluorescent methods [70].

The use of copper ions in click reactions that underlie nanopore-based detection has become widespread in recent times. Recently, a work was published on the determination of HIV-1 p24 in clinical samples, which used the principles of this reaction for nanopore-based detection of the antigene. Even though copper ions were not determined in this work, the work is of great significance in the field of nanopore-based detection of biomolecules [71].

**Figure 3:**
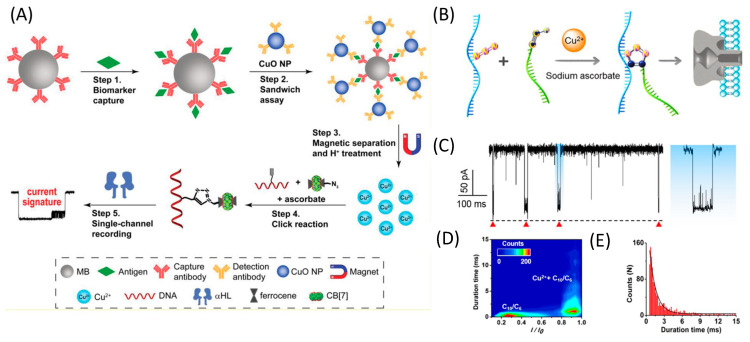
Nanopore pulse detection of Cu^2+^ ions using click chemistry. (**A**) Scheme of the strategy for the detection of biomarkers based on sandwich assay. Reproduced from [72]. Copyright 2018 John Wiley and Sons (**B**) Scheme of copper ion detection with individual ssDNA fragments (C_10_ and C_6_), one internally modified with azide and the other end-modified with alkyne. (**C**) Current trace recorded in the presence of Cu^2+^ and monitored at +150 mV in 1 M KCl, with red triangles representing signature events. (**D**) Contour plot for Cu^2+^ reaction products and ssDNA. (**E**) Duration histogram Reproduced from [34]. Copyright 2019 American Chemical Society.

Earlier, a nanopore technology was developed for the detection of alpha-fetoprotein (a cancer biomarker in human blood) (Figure 3A). The DNA probe was split into two parts, one was the alkyne-modified single-stranded DNA (ssDNA) and the other was a complex between azide-containing ferrocene and cucurbit[ 7 ] uril. The authors showed that the frequency of translocation was directly proportional to the concentration of copper ions in solution, and foreign metal ions did not affect the frequency of translocations [72].

In a recent study, a similar technique was employed for the detection of copper ions. As depicted in Figure 3B, the probe was divided into two individual ssDNA fragments, one internally modified with azide and the other end-modified with alkyne. The click reaction induced the fusion of these two fragments, thereby generating a branched DNA probe. As this branched DNA traversed the α-hemolysin nanopore, it produced highly distinctive current events. The method exhibited a good linear correlation between the frequency of events and the logarithm of Cu^2+^ concentration within a range of 100 pM to 5 μM (LOD 67 pM) [34]. In this study, the authors developed a method that demonstrated comparable or superior detection limits to previous methods. The practicability of this approach was evaluated by analyzing human serum and tap water samples that had been enriched with copper ions. The nanopore-based sensor platform was found to be suitable for analyzing complex samples, with recovery rates of 89.5–105.3% and 90.2–103.8% in serum and tap water, respectively.

Nanopore technology has a remarkable ability to detect copper ions with high sensitivity, thanks to specific molecules for chelating copper ions designed for this purpose. While this technology has been most effectively used in aqueous solutions, its use in living organisms is limited by the challenge of creating a minimally invasive sensor with a biological nanopore. However, it is possible that more advanced biological nanopores will be developed in the future, which could accurately and sensitively measure copper ion concentrations within cells and tissues. This breakthrough could lead to new opportunities for investigating copper’s role in biological processes and developing innovative diagnostic and treatment methods for diseases related to copper metabolism disorders.

### 3.2. Ionic Current Rectification in Nanopores Technique

In recent years, a lot of research have been published using the ICR detection technique, including summarizing review articles [39]. However, there are few studies with the main goal of detecting Cu^2+^. Therefore, in this section, we will focus on describing existing research from previously unconsidered points of view and discuss a new one.

It is noteworthy that half of the studies using the ICR technique for Cu^2+^ detection developed a sensor based on a macropipette with a nanopore at the sharp tip. Using a pipette with a nanopore (further termed “nanopipette”) as a base for further sensor development has several features. Firstly, the nanopipette is an easy-to-handle product. All manipulations during sensor fabrication are performed with its macro part, which does not require high-precision techniques and tools. Secondly, the nanocapillary sensor can be easily combined with a micromanipulator and an optical microscope to carry out measurements with high spatial resolution [35]. The final advantage is the possibility of combination with other research methods. For example, in recently published research, authors used a double-barrel nanopipette sensor. One channel was used to detect pH changes using the ICR technique, while the other one was used for topographical imaging using SICM. Thus, combining two strong techniques allowed real-time simultaneous 3D topographical imaging and pH monitoring of living cancer cells [73].

**Figure 4:**
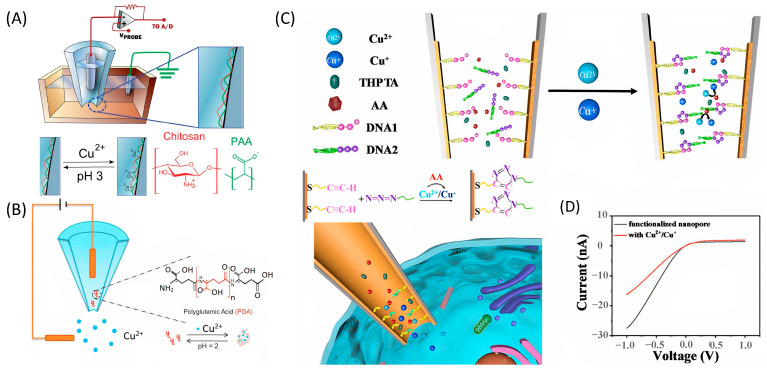
Schematic representation of the Cu^2+^ detection principle using nanopipette sensors and the ICR detection technique. (**A**) Electrochemical configuration and reversible binding of Cu^2+^ ions on the chitosan/PAA nanopipette. Reproduced from [74]. (**B**) Single glass capillary nanopore-based sensing platform with non-immobilized PGA probes. Reproduced from [75]. Copyright 2015 Elsevier Copyright 2011 American Chemical Society (**C**) Alkyne-end ssDNA-functionalized G-nanopore for Cu^2+^ or Cu^+^ detection in single cells based on click chemistry. (**D**) Typical I–V curves of the functionalized AG-nanopore for Cu^2+^ or Cu^+^ detection in a single cell. Reproduced from [35]. Copyright 2022 American Chemical Society.

One of the first studies aimed at the detection of metal ions (Cu^2+^ predominantly) using the ICR technique was the study by Paolo Actis et al. [74]. The authors used a nanopipette sensor functionalized with chitosan and poly(acrylic acid) (Figure 4A). The layer-by-layer modification technique consisted of forming layers of chitosan and poly(acrylic acid) by sequentially immersing a nanopipette in the appropriate solutions and applying a sinusoidal voltage to achieve real-time control of the dynamic process. The developed sensor showed a linear detection range from 4 to 100 µM. Thus, a non-selective ICR nanopipette sensor for the detection of metal ions was developed, which laid the foundation for subsequent studies aimed at the detection of Cu^2+^ with a wide range of applications.

The next published study had the main goal of achieving stable detection of Cu^2+^ with high selectivity [75]. The authors used PGA as a non-immobilized probe, which was highly specific to Cu^2+^ in aqueous solution at pH 7–8 (Figure 4B). Great attention was paid to the variation of asymmetric salt gradients that significantly impacted the sensitivity of the sensor. The average linear range of sensor detection was 7.5 to 60 µM with an LOD of ~1.05 µM. The selectivity of the sensor was investigated by evaluating the effect of non-target metal ions on the rectification factor. It was established that the influence of interfering metal ions was insignificant and the sensor regeneration (in buffer pH 2) was ~95%. For the first time, in this work, the practical application of a nanopipette ICR sensor for the investigation of real samples was shown. Cu^2+^ was detected in real samples of grape wine and industrial water, confirming numerous previous selectivity tests.

The detection of Cu^2+^ was slightly touched on in the literature [76]. The authors used a nanopipette modified with tannic acid to provide the polyphenolic functional groups. The main feature of this work was the different current rectification after binding of different valent metal ions with the sensor surface. Such a strategy made it possible to successfully detect trivalent ions with ultrahigh sensitivity (LOD of Fe^3+^ was 10^−15^ M) and also divalent ions (such as Cu^2+^) with lower sensitivity.

The final study with a nanopipette-based sensor was the quantitative determination of Cu^+^/Cu^2+^ inside single cells reported by Hu [35]. The authors fully realized the suitability of nanopipette sensors for low-invasive measurements of biological systems [77]. In the sensing scheme, the thiolated ssDNA with an alkynyl end was first functionalized onto a gold-modified glass nanopipette. Copper ions catalyzed the cycloaddition reaction between an azide-end single-stranded DNA (ssDNA) and an alkynyl-functionalized glass nanopipette, which caused obvious rectification changes of the sensor (Figure 4C,D). The selectivity of the nanopipette sensor towards copper ions was high. However, the presence of ion mixtures significantly complicated the detection process. Using the developed sensor, the authors conducted correlation studies to assess the relationship between ROS generation and copper accumulation inside single HepG2 cells (human liver carcinoma-derived). It was confirmed that a higher Cu^2+^/Cu^+^ ratio in single cells led to an increase in ROS generation.

A non-nanopipette-based sensor was presented by Jyh-Ping Hsu. It was an interesting case in which a conical PET nanopore surface was used with the ICR technique for detection [78]. The authors used PET membranes functionalized by 18-crown-6 for the detection of trace levels of heavy metal ions (Pb^2+^ predominantly). The main part of the study was modeling ionic transport in nanochannels under different conditions affecting electroosmotic flow. The developed conical nanochannels showed the possibility of sensing Pb^2+^ or/and Cu^2+^ with an LOD of 0.05 and ~10 µM, respectively. Thus, the authors emphasized that the conical PET nanopore with the ICR technique could be potentially used for the quantitative analysis of human blood in which the concentration of metals is strictly controlled.

An extraordinary approach to the determination of Cu^2+^ was developed by the Tietze group [79]. The authors used the 5/6-FAM-Dap-β-Ala-His fluorescent peptide for selective binding of Cu^2+^ ions. The fluorescent peptide was simultaneously used as a PET nanopore modifier and an independent fluorescent probe. Thus, the Cu^2+^ detection mechanism was based on the ICR technique and fluorescence quenching upon binding between Cu^2+^ and the peptide. The linear ranges of this sensor were from 1 to 100 μM (for fluorescence quenching) and from 10 fM to 0.1 μM (for ICR technique), which was a record among the described research in this section.

Finally, Zhao et al. used a nanochannel array of porous anodic alumina for Cu^2+^ detection [80]. The authors modified nanopores with PGA, which was used as a high-selectivity modification agent in other described study [75]. In contrast to the previous study, the use of an array of nanopores with a highly enhanced magnitude of the ion current made it possible to significantly increase the sensitivity of the technique. The LOD was 0.1 fM. The practical application of the nanochannel array was confirmed by measuring the concentration of Cu^2+^ in real blood samples.

Thus, in the first half of this section we demonstrated the evolution of nanopipette sensors aimed at Cu^2+^ detection using the ICR detection technique. The first study demonstrated the potential application of this type of sensor followed by research with real applications, including investigation of complex biological systems. The investigation of biological systems with nanopipette sensors based on the ICR detection technique is an indisputable advantage over the translocation technique, even despite the significantly smaller detectable range of concentrations. Further evolution of this scientific field, in our opinion, should include the search for solutions that can increase sensitivity and selectivity in the presence of several types of non-target ions. This will allow more accurate measurements of real samples, in which the presence of several types of ions is often commonplace. In addition to the above, we have described research using PET nanopores or nanopore arrays of porous anodic alumina. Notably, interesting strategies have been applied to significantly increase sensitivity, such as the simultaneous use of ICR and fluorescence quenching or the use of a nanochannel array. We summarized of strategies for copper detection in Table 1.

## 4. Conclusions and Outlook

In conclusion, we reviewed several works devoted to the detection of copper ions using various techniques with nanopores. In this review, we have focused exclusively on the detection of copper ions, since copper is an important element in the body and is involved in a huge number of biochemical processes. First, we considered a strategy for detecting copper ions based on the registration of events characteristic of the passage of complexes with copper through a nanopore. In most of these works, biological nanopores based on hemolysin were used, while the number of works with solid-state nanopores is extremely small. Nevertheless, this type of nanopore is the most suitable for the development of portable sensors. Immobilization of the binding site in a solid pore is very difficult; therefore, the development of a strategy for detecting copper ions using this type of nanopore can be carried out by introducing additional highly specific molecular probes capable of chelating copper ions. Another direction of development in this area is the development of high-performance nanopore-based sensing, namely the creation of an array of nanopores to improve the sensitivity and accuracy of determining metal concentration.

In addition, nanopore-based sensors can be integrated into microfluidic devices for automated and high-throughput analysis. Microfluidic devices can be used to precisely control the flow of samples through nanopores, allowing fast and efficient analysis of large volumes of samples. Integrating nanopore-based sensors into microfluidic devices can also reduce the volumes of samples and reagents required for analysis, making analysis more economical and environmentally friendly.

Secondly, a detection strategy based on current rectification in the nanopore was considered. Fundamentally two types of nanopores were used in the reviewed studies. In the case of using a nanopipette, there is a clear trend from the first works with the development of non-selective sensors to research aimed at the intracellular determination of Cu^2+^. We expect that researchers will focus on expanding the use of nanopipettes as an easy-to-use stable tool for measurement of in vitro/in vivo biological systems. In the second case of using various types of nanopores with the ICR technique, it is obvious that nanopores will be integrated into arrays and devices in the future or combined with other methods of analysis. Thus, it will be possible to significantly increase the sensitivity of the current rectification technique. Nanopore technology offers a promising platform for the sensitive and selective detection of copper ions in a variety of samples. The ability to detect copper ions in real time and at low concentrations with nanopore-based sensors could be important in various fields, such as environmental monitoring, food safety, and medical diagnostics.

## Figures and Tables

**Table 1:** Summarized table of strategies for copper detection.

Analyte	Type of Nanopore	LOD	Linear Range	Sensing Principe	Used Ligand	Refs.
Cu^2+^	α-hemolysin	40 nM	-	RPS	Histidine-peptide	[33]
Cu^2+^/Cu^+^	α-hemolysin	67 pM		RPS	DNA	[34]
Cu^2+^	α-hemolysin	12 nM	0.08–20 µM	RPS	Polyamine-decorated cyclodextrins	[60]
Cu^2+^	α-hemolysin	-	-	RPS	Carboxymethyl-β-cyclodextrin	[61]
Cu^2+^	α-hemolysin	16 nM	0.03–1.0 µM	RPS	TPPS	[62]
Cu^2+^	α-hemolysin	1 ppm	-	RPS	Modified silicon dioxide nanoparticles	[64]
Cu^2+^	α-hemolysin	-	-	RPS	Prion peptide	[65]
Cu^2+^	Glass nanopipette	-	4–100 µM	ICR	Chitosan and poly(acrylic acid)	[74]
Cu^2+^	Glass Nanopipette	1.05 µM	7.5–60 µM	ICR	PGA	[75]
Cu^2+^	Glass Nanopipette	-	~1–40 µM	ICR	Thiolated ssDNA with an alkynyl end, azide-end single-stranded DNA	[35]
Cu^2+^	PET nanochannel	-	10–500 µM	ICR	18-crown-6	[78]
Cu^2+^	PET nanochannel	18 nM (Fluorescence quenching)	10 fM–0.1 μM (ICR technique) 1–100 μM (Fluorescence quenching)	ICR and Fluorescence quenching	5/6-FAM-Dap-β-Ala-His fluorescent peptide	[79]
Cu^2+^	Nanochannel array of porous anodic alumina	0.1 fM	-	ICR	PGA	[80]

## Abbreviations

βCD	β-cyclodextrin
AAS	atomic absorption spectroscopy
AES	atomic emission spectroscopy
APTES	(3-aminopropyl)triethoxysilane
CMβCD	carboxymethyl-β-cyclodextrin
EDTA	ethylenediamine tetraacetic acid
ICP-MS	inductively coupled plasma mass spectrometry
ICR	ion current rectification
LOD	limit of detection
PGA	polyglutamic acid
PrP	prion peptide
RPS	resistive pulse sensing
TPPS	5,10,15,20-tetrakis(4-sulfonatophenyl)-porphyrin
